# Intraoral Scanning Enables Virtual-Splint-Based Non-Invasive Registration Protocol for Maxillofacial Surgical Navigation

**DOI:** 10.3390/jcm13175196

**Published:** 2024-09-02

**Authors:** Max Wilkat, Leonardo Saigo, Norbert Kübler, Majeed Rana, Felix Schrader

**Affiliations:** 1 Department of Oral and Maxillofacial Surgery, Heinrich Heine University Hospital Düsseldorf, Moorenstraße 5, 40225 Düsseldorf, Germany; 2 Department of Oral and Maxillofacial Surgery, National Dental Centre Singapore, 5 Second Hospital Ave., Singapore 168938, Singapore

**Keywords:** surgical navigation, computer-assisted planning, intraoral scanning, navigational registration, maxillofacial surgery

## Abstract

**Background/Objectives:** Surgical navigation has advanced maxillofacial surgery since the 1990s, bringing benefits for various indications. Traditional registration methods use fiducial markers that are either invasively bone-anchored or attached to a dental vacuum splint and offer high accuracy but necessitate additional imaging with increased radiation exposure. We propose a novel, non-invasive registration protocol using a CAD/CAM dental splint based on high-resolution intraoral scans. **Methods**: The effectiveness of this method was experimentally evaluated with an ex vivo 3D-printed skull measuring the target registration error (TRE). Surgical application is demonstrated in two clinical cases. **Results**: In the ex vivo model, the new CAD/CAM-splint-based method achieved a mean TRE across the whole facial skull of 0.97 ± 0.29 mm, which was comparable to traditional techniques like using bone-anchored screws (1.02 ± 0.23 mm) and dental vacuum splints (1.01 ± 0.33 mm), while dental anatomical landmarks showed a lower accuracy with a mean TRE of 1.84 ± 0.44 mm. Multifactorial ANOVA confirmed significant differences in TRE based on the registration method and the navigated level of the facial skull (*p* < 0.001). In clinical applications, the presented method demonstrated high accuracy for both midfacial and mandibular surgeries. **Conclusions**: Our results suggest that this non-invasive CAD/CAM-splint-based method is a viable alternative to traditional fiducial marker techniques, with the potential for broad application in maxillofacial surgery. This approach retains high accuracy while eliminating the need for supplementary imaging and reduces patient radiation exposure. Further clinical trials are necessary to confirm these findings and optimize splint design for enhanced navigational accuracy.

## 1. Introduction

Surgical navigation, first described in the 1990s, has become well established in the field of maxillofacial surgery [1,2,3,4]. This technology facilitates the real-time localization of anatomical structures within corresponding DICOM datasets, enhancing intraoperative orientation and enabling the identification and preservation of critical anatomical features. For instance, surgical navigation aids in the accurate identification and protection of vital structures, thereby increasing surgical safety. Additionally, preoperative virtual planning datasets can be correlated intraoperatively, which is crucial in oncological surgery for identifying preoperatively segmented resection margins to ensure accurate resection volume delineation during surgery [5,6,7]. This technique also supports trajectory-guided, minimally invasive biopsy procedures [8]. In trauma surgery, surgical navigation enables the repeatable intraoperative assessment of repositioning outcomes during primary and secondary fracture treatments, which can be beneficial in cases like complex zygomatic fractures [9,10]. Here, navigation can help restore facial symmetry by facilitating the real-time querying of the virtually mirrored contralateral side for correct sagittal and transverse dimensional reconstitution. Even in cases of bilateral fracture patterns, where virtual reduction facilitates a virtual surgical plan to aim for, surgical navigation allows intraoperative checking for correct plan transfer [11]. Furthermore, navigation assists in verifying the correct positioning of patient-specific implants, especially in complex areas like the posterior orbit, thereby enhancing safety and accuracy outcomes in orbital reconstructions [12,13].

Since its inception, numerous advancements have been made in both the technical and clinical applications of surgical navigation [14,15,16,17]. The essential components of surgical navigation that dictate its accuracy include tracking, referencing, and registration. For tracking, there are two techniques which are most often used: optical and electromagnetic tracking [18]. Optical tracking relies on the visibility of the tracked object by the navigation system’s cameras, often using infrared-reflective spheres, which requires a constant line of sight to facilitate high accuracy. Electromagnetic tracking, on the other hand, allows for the use of smaller sensors that do not require a constant direct line of sight. However, its accuracy can be reduced due to ferromagnetic interference, which can occur during intraoperative use. Skeletal referencing is considered the most accurate for bone navigation due to the absence of soft tissue shift as long as the referenced bone is integral or fixed to the navigated bone. Non-invasive headband referencing is used in ENT or pediatric surgery for certain indications but presents limited accuracy in bone surgery [19,20,21].

Various registration processes have been described for surgical navigation [22]. Unlike other surgical disciplines where shifts between preoperative imaging and intraoperative anatomy (e.g., due to patient positioning in spine surgery or brain shift in neurosurgery) can necessitate repeated intraoperative imaging [23,24], maxillofacial surgery often avoids this, reducing technical and financial burdens. Consequently, the registration process is crucial in maxillofacial surgery for ensuring intraoperative accuracy with reference to the preoperative dataset and virtual planning [25]. The target registration error (TRE) can be used to quantify the registration accuracy as it measures the difference between the actual and the expected position of a specific target point after registration.While a surface scan-based registration process of the facial soft tissue can be fast-forwarded and is often used in diagnostic-driven surgeries in the ENT specialty, it might offer limited TRE accuracies of ≈2 mm due to soft tissue shifting not meeting the levels needed for maxillofacial bone surgery. Therefore, a point-based registration process is widely considered as the standard when it comes to maxillofacial traumatology or ablative surgery [25]. With the use of bony anatomical landmarks, the point-based registration can usually achieve TRE values of below 2 mm, potentially enhancing the accuracy levels compared to the surface-based method [26]. The invasive placement of radio-opaque fiducial markers, such as bone-anchored titanium osteosynthesis screws, enhances the accuracy to a TRE of approximately 1 mm because the screw heads can be precisely targeted and reproducibly localized with the navigation probe [25]. This method requires additional preoperative imaging (at least CBCT or CT), which can be matched to the baseline dataset through voxel-based matching [27]. However, the invasiveness of this approach is a drawback. A dental splint is a non-invasive alternative in patients with sufficient and stable dentition, offering accuracy comparable to bone-anchored markers without surgical invasiveness [28]. The standard protocol involves creating a dental vacuum-formed splint in a dental laboratory based on alginate impression-driven plaster models. Titanium osteosynthesis screws are polymerized into the splint at a minimum of four vestibular positions, typically alternately angled in the cranio–caudal axis to maximize the navigation accuracy. Certain modifications facilitate setups that enable navigation in the mandible as well [29]. However, metallic restorations in the dental region can obscure the screw heads in the DICOM dataset due to artifact formation leading to decreased registration accuracies [30]. Both bone-anchored screws and screws attached to a dental splint require additional imaging to serve as radio-opaque fiducial markers in the DICOM dataset, resulting in further radiation exposure for the patient [27].

We present a modern protocol for fabricating a dental splint for registration during surgical navigation in the maxillofacial region, eliminating the need for additional radiation-based imaging. Only an intraoral surface scan of the dentition using a commercial intraoral scanner and a software-based matching algorithm are required. We hypothesize that the registration accuracy with this new protocol is equal to conventional point-based registration methods using radio-opaque fiducials. The registration accuracy was experimentally evaluated through TRE determination in an ex vivo skull model. Moreover, this new registration protocol for surgical navigation has been successfully used in clinical applications. We present two clinical cases to illustrate the clinical workflow for surgical navigation in the midfacial and mandibular regions, emphasizing its relevance in achieving precise surgical outcomes.

## 2. Materials and Methods

This study was conducted in accordance with the ethical principles of the Declaration of Helsinki. Approval for the study was granted by the Ethics Committee of Heinrich Heine University Düsseldorf (study number: 2023–2716). Informed consent was obtained from all patients.

### 2.1. Protocol for Registration Splint Fabrication and Usage in Surgical Navigation

A standardized protocol for the computer-assisted design and manufacturing of dental registration splints involving intraoral scans of the dentition as the basis for correct integration into the dataset to be registered has been established for usage in maxillofacial surgical navigation. Details are described in the following.

#### 2.1.1. Virtual Splint Generation

For virtual surgical planning, data collection comprised a cone beam computed tomography (CBCT) or a computer tomography (CT) scan capturing the patient’s facial skull. If navigation in the mandible is required, the scans should capture the patient’s habitual occlusion with a central fossa–condyle relationship. A wax bite may be used to achieve correct habitual occlusion if the scan is scheduled and there is no need to use a pre-existing scan. Additionally, the intraoral scans of both the upper and lower dentitions are taken (TRIOS 3 intraoral scanner, 3Shape A/S, Copenhagen, Denmark), along with a third surface scan merging both dentitions in the patient’s habitual occlusion relationship if navigation in the mandible is desired.

**Figure 1:**
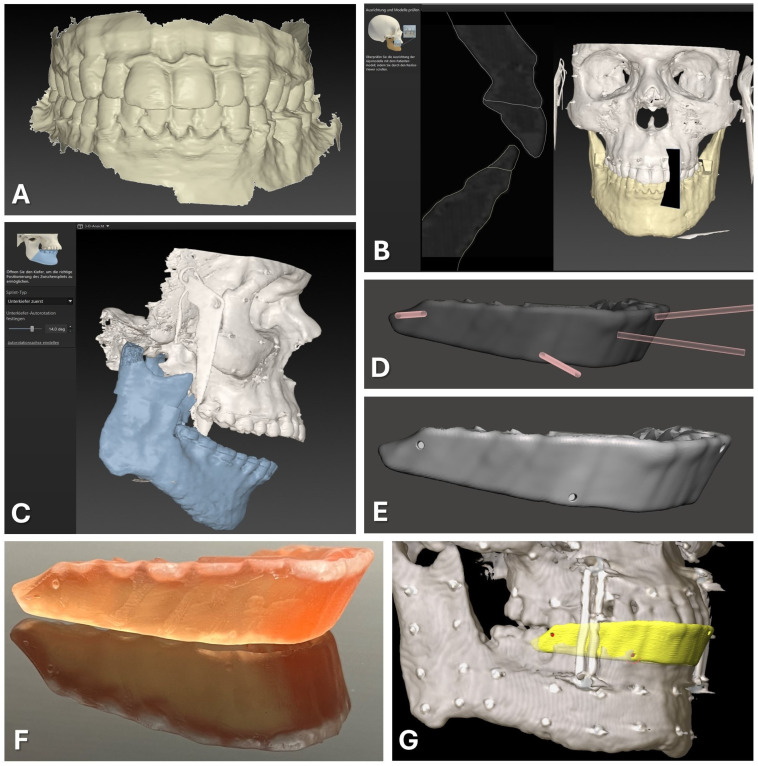
CAD/CAM of registration splint. Steps 1 to 7 are displayed as described in the text in detail, beginning with the intraoral scan of the upper and lower dentition (**A**). The second step of semi-automatic matching through the software IPS Case Designer is displayed (**B**). Next, an intermediate splint is generated following the mandible-first protocol with a high degree of mandibular autorotation of >10° (**C**). Modification of the splint design through Boolean subtraction is depicted using the freeware Autodesk Meshmixer (**D**). Final splint design (**E**) can be manufactured via 3D-printing using transparent surgical guide resin in orange color (**F**). For surgical navigation, the STL of the registration splint is imported into the Brainlab software iPlan 3.0.5 along with the DICOM data, and four virtual registration landmarks (red) can be positioned at the bottom of the indentations of the vestibular surface of the splint (yellow), perfectly aligning with the upper dentition (**G**).

The IPS Case Designer software (V2.3.5.2, KLS Martin, Tuttlingen, Germany), a virtual planning software dedicated to orthognathic surgical procedures, is used for the raw splint design. After aligning the DICOM data according to the Frankfurt horizontal plane and midfacial sagittal plane, the software’s algorithm semi-automatically segments the facial skull. The user approves the threshold for hard and soft tissue. Next, the intraoral scans of the upper and lower dentition are loaded in STL format and semi-automatically matched to the DICOM dataset after the user indicates five mandatory landmarks (right and left condyle, mesio-vestibular cusp of the first upper molar right and left, and upper incisal point). The matching result is assessable in the sagittal or any other plane and requires user approval (see Figure 1B). This step yields a virtual reconstruction of the facial skull, integrating high-resolution geometry information at the occlusion surfaces of the teeth taken from the intraoral scans.

Subsequently, the software outlines the osteotomy lines for procedures such as Le Fort I osteotomy and bilateral sagittal split osteotomies (BSSOs) and plans jaw movements. The exact location of the osteotomy lines and the degree of movement are not relevant for generating the desired dental splint usable for surgical navigation. After the planning phase is completed, the software facilitates the design of occlusal splints for intermediate and final positions of the repositioned maxillary and mandibular segments, which can be exported in STL file format.

For surgical navigation, a dental splint is required that aligns with the original position of the maxilla and provides sufficient vertical height to serve as a registration splint. This desired splint design corresponds to the intermediate splint used in the mandible-first protocol, featuring a wide-open locked bite with at least 10° of autorotation of the mandible around the intercondylar axis (see Figure 1C). Thus, the intermediate splint of the mandible-first protocol was generated following the software‘s algorithm and exported in STL file format for further modification.

#### 2.1.2. Virtual Splint Modification for Usage for Registration

The exported intermediate splint following the mandible-first protocol was saved in STL file format and underwent further digital modifications. Utilizing Autodesk Meshmixer freeware (Autodesk Research, San Francisco, NC, USA), circular indentations with a diameter of 1.5 mm and a depth of approximately 3 mm were created on the vestibular face of the splint by performing Boolean subtraction between the splint and a cylindrical object of the mentioned diameter (refer to Figure 1D,E). These indentations facilitated the precise intraoperative positioning of the Brainlab surgical navigation system’s probe, featuring a pointed end with an approximate diameter of 1 mm. 

Four indentations were strategically placed at the positions of the first upper molars (left and right) and the canine teeth (left and right). These indentations were evenly distributed along the vertical axis, with two positioned above and two positioned below the occlusion plane. This distribution of indentations is necessary to improve the accuracy of the surgical navigation as a skew quadrilateral is put up in space between the four indentations covering the center of the area of interest for the navigated procedure.

#### 2.1.3. Splint Fabrication

The modified splint design (see Figure 1) was imported into the Preform software (version 3.27.1, Formlabs Inc., Somerville, MA, USA) for additive 3D printing preparation. Support structures were added without obstructing occlusal surfaces or navigational indentations. Utilizing the surgical guide resin V1 in the Form 2 3D printer (Formlabs, version 3.27.1, Formlabs Inc., Somerville, MA, USA) and a slice thickness of 0.05 mm, the 3D printing process was executed. After completing 3D printing and post-processing as per the manufacturer’s protocol, navigational indentations were marked using a pen.

#### 2.1.4. Navigational Planning 

For the preparation of surgical navigation, the DICOM data of the same CBCT or CT scan which was used for the registration splint fabrication was imported into iPlan CMF 3.0.5 (Brainlab AG, Munich, Germany). The dataset was aligned according to the Frankfurt horizontal plane and midfacial sagittal plane. Before importing the exported STL file of the modified registration splint, the file had to be rotated by 180° around the *z* axis to align with the world axis of the Brainlab system. The rotation of the STL file was performed using Geomagic Freeform (version 2020.1.1, Oqton, Los Angeles, CA, USA). The rotated STL file was imported into the iPlan CMF software. Four registration points were set on the surface of the bottom of the four indentations on the modified registration splint (see Figure 1G). During the intraoperative setup of surgical navigation utilizing the skull reference array, the manufactured registration splint is placed onto the upper dentition, and the virtually set navigation points are selected with the navigation probe to complete the registration process.

### 2.2. Skull Model Experiment for Evaluation of Registration Methods 

To evaluate the accuracy of the new registration protocol, it was compared to other methods of point-based registration for surgical infrared-based navigation in an experimental set-up involving an ex vivo 3D-printed skull. The following methods of registration were compared:

1. Method 1: bone-anchored MMF stainless steel screws;
2. Method 2: dental registration splint manufactured by vacuum thermoforming with polymerized osteosynthesis titanium screws;
3. Method 3: dental CAD/CAM registration splint based on intraoralscan matching and 3D resin printing (new protocol);
4. Method 4: dental landmarks.

#### 2.2.1. Skull Printing

The skull models of one randomly selected patient surgically treated for OSAS with an Angle Class I occlusion were fabricated using a Dimension Elite 3D-Printer (StrataSys, Eden Prairie, MN, USA). These models were generated based on the preoperative DICOM data obtained from computer tomography (CT) which had been part of the preparation for maxillomandibular advancement surgery. The atlas-based segmentation of the facial skull including midface/skull base and mandible which were exported as two independent STL-files was performed using iPlan CMF 3.0.5 (Brainlab AG, Feldkirchen, Germany).

**Figure 2:**
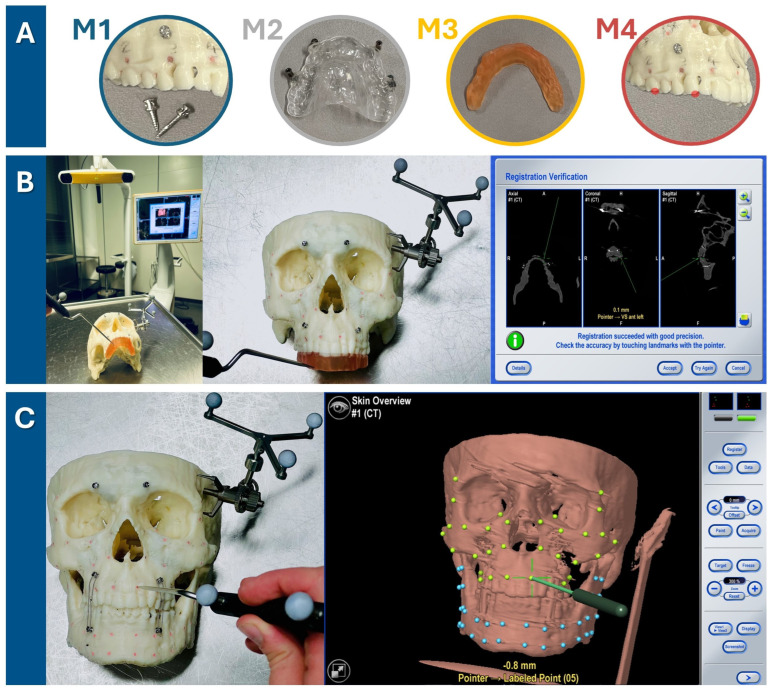
Target registration error verification in three steps. (**A**) Firstly, one of four registration methods was chosen (M1: bone-anchored screws, M2: dental vacuum-splint with screws, M3: dental CAD/CAM splint, M4: dental landmarks). (**B**) For each run-through, the registration process for one method (here, the method M3 is shown as an example) was performed. The setup is illustrated on the left, featuring the navigation system’s infrared camera and monitor in the background. The detailed view in the middle image shows the 3D-printed skull with the skull reference array attached to the temporal bone and the upper dentition covered by the CAD/CAM registration splint. As the observer points to the four indentations on the registration splint, the system tracks the registration. Successful registration is confirmed by checking alignment at the monitor, either by pointing to the registration landmarks or anatomical landmarks. (**C**) Following successful registration, the mandible was secured with wires to the MMF screws in habitual occlusion. Using the navigation probe, the observer pointed perpendicularly to the camera view axis at each gutta-percha point. The system then displayed the estimated distance between the probe tip and the gutta-percha point, defined as the target registration error (TRE) value. TRE values were recorded twice for each of the 64 guttapercha points (32 green targets in the midface and 32 blue targets in the mandible), with the process repeated three times for each registration method—twice by the first observer and once by a second observer.

To establish reference points for measurements, holes with a diameter and depth of approximately 1 mm were drilled into the skull and filled up with plastified gutta-percha (Dentsply, Konstanz, Germany). In total, 32 gutta-percha-filled holes were created so that they were symmetrically distributed across both the midfacial skull and mandible, resulting in a total of 64 gutta-percha-filled holes (see Figure 2).

Subsequently, the skull model with the mandible fixed to the skull in habitual occlusion using wires across MMF screws underwent CBCT examination. For the first two registration methods, namely “bone-anchored screws” and “dental vacuum splint with screws”, the registration markers were applied to the skull and a second CBCT scan was obtained.

#### 2.2.2. Navigation Planning Using Different Registration Methods

The obtained data were processed for experimental navigation on a 3D-printed skull using navigation software (iPlan 3.0.5, Brainlab AG, Feldkirchen, Germany). For the first two registration methods, the matching of a second CBCT scan showing the radio-opaque fiducial markers was necessary using the software’s voxel-based matching algorithm. The digital marking of the four registration points was performed for all four registration methods:

1. Marking of the 4 screw heads of the bone-anchored screw on the second-matched CBCT scan across the midface (two screw heads in the maxillary alveolar process, two screw heads in the fronto-orbital area);
2. Marking of the 4 screw heads of the dental navigation splint on the second matched CBCT scan;
3. Marking of the 4 indentations on the CAD/CAM registration splint loaded as an STL file;
4. Marking of 4 dental landmarks as visualized in the DICOM dataset: tip of the mesio-vestibular cusp of the first upper molar right and left, tip of the upper canine right and left.

The digital marking of the 64 gutta-percha-filled radio-opaque holes as target measurement points completed the digital planning for the experimental navigation procedure.

#### 2.2.3. Target Registration Error Verification 

The Brainlab Curve Dual Display navigation system (Brainlab AG, Feldkirchen, Germany) was utilized for this experiment. The skull reference array was affixed with a screw to the left temporal bone of the 3D-printed skull (see Figure 2). One of the four registration methods was executed by marking the respective landmarks until the system recorded their positions and accepted the registration procedure. Thereafter, the mandible was fixed with wires over MMF screws following the habitual occlusion to also allow measurement in the mandible. The target registration error (TRE) was measured by precisely aligning the navigation probe‘s tip with each of the 64 gutta-percha-filled holes (32 in the midface, 32 in the mandible) and recording the minimal distance from the probe to the corresponding digital target measurement point, as indicated by the navigation system (see Figure 2). As pivoting movements affected the displayed minimal distance, the smallest possible value was recorded for each of the 64 measurement points, which was typically achieved by holding the pointer perpendicular to the viewing axis of the infrared camera of the navigation system. Two measurement values per gutta-percha measurement point were recorded during one run-through of one registration method. The total TRE measurement process for each of the four registration methods was performed three times (twice by the first observer and once by the second observer) to assess intra- and interobserver reliability.

#### 2.2.4. Statistics 

Data collection and storage was carried out using Excel spreadsheets (Excel 14.0, Microsoft Corporation, Washington, DC, USA). The statistical evaluations were carried out with the software IBM SPSS Statistics (IBM Corp. Released 2023. IBM SPSS Statistics for Macintosh, Version 29.0.1. IBM Coro, Armonk, NY, USA).

The mean and standard deviation of the primary outcome parameter target registration error for each registration method was calculated and plotted against the distance to the center of registration. The Shapiro–Wilk normality test and Levene test of variance were performed. A comparison between groups was evaluated using a multifactorial analysis of variance with repeated measurements (ANOVA) followed by Bonferroni’s post hoc analysis. The first factor was the registration method used (M1: bone-anchored screws, M2: dental vacuum splint with screws, M3: dental CAD/CAM splint, M4: dental landmarks); the second factor was the side (right, left); the third factor was the region (midface, mandible); and the fourth factor was the level of the targets (0: ramus; 1: inferior mandibular border; 2: mandibular alveolar process; 3: maxillary alveolar process; 4: maxillary sinus; 5: zygoma and inferior orbital rim; 6: lateral orbital rim and posterior orbit). The dependent variable was the target registration error (TRE). The interactions between factors were further analyzed using Student’s *t*-test. *p*-values of *p* < 0.05 were considered significant.

Intra- (both run-throughs of observer (1)) and interobserver (first run-through of observer 1 and run-through of observer (2)) reliability were assessed for each method M1–M4 by calculating the ICC (Intraclass Correlation Coefficient) and their 95% confidence intervals based on a 2-way mixed-effects model with absolute agreement. ICC values were interpreted according to Koo et al. [31] with values ranging from 0 to 1, where values below 0.5 are considered poor, values between 0.5 and 0.75 are considered moderate, values between 0.75 and 0.90 are considered good, and values above 0.90 are considered excellent in terms of agreement/reliability. 

### 2.3. Surgical Cases

Two cases are presented to demonstrate the clinical application of the new registration protocol which was applied as described above to facilitate surgical navigation in the midfacial and mandible region. Clinical findings, surgical plan and procedure, as well as outcome are presented in the results section.

## 3. Results

### 3.1. TRE Evaluation in Ex Vivo Skull Model for Comparison of Different Registration Methods

**Figure 3:**
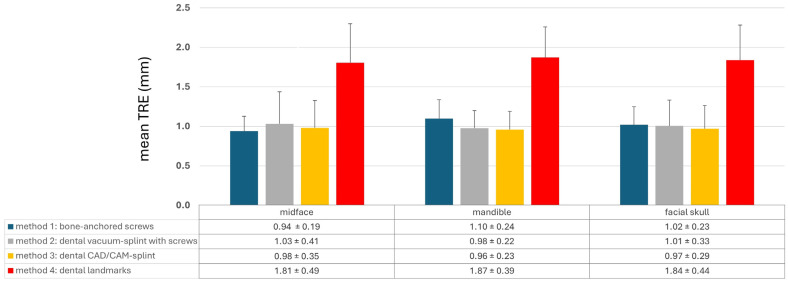
Mean target registration error (TRE) for each registration method M1–M4 separated into the region of the midface, of the mandible, and the whole facial skull. Whiskers show the standard deviation.

The different registration methods resulted in varying TREs. The mean TREs for the respective methods are shown in Figure 3. The TREs for M1–M3 across the entire facial skull were approximately 1 mm (M1: 1.02 ± 0.23 mm; M2: 1.01 ± 0.33 mm; M3: 0.97 ± 0.29 mm), whereas method M4 was significantly less accurate with a mean TRE of 1.84 ± 0.44 mm. For method M1, the mean TRE decreased in the midface compared to the entire facial skull, while it slightly increased for the other three methods. This can be attributed to the different distribution of registration markers and thus the different locations of the registration center, which for M1 was below the tip of the nasal bone, while the registration center for M2–M4 was at the level of the occlusal plane. Consequently, the mean TRE for M2–M4 was lower in the mandible compared to the midface, whereas for method M1, it was the opposite.

**Figure 4:**
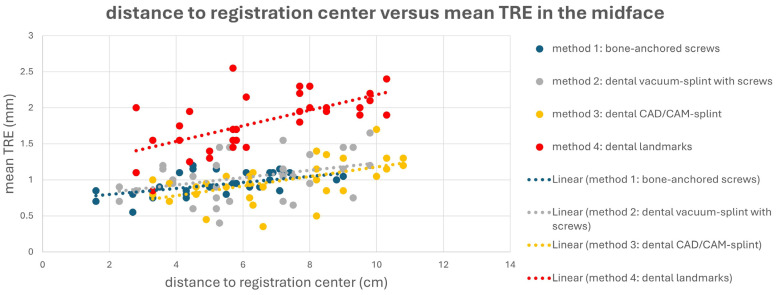
Point cloud plotting distance of the registration center in cm (*x* axis) versus the mean target registration error (TRE) in mm (*y* axis) in the midface for each registration method M1–M4. The regression line is displayed as dotted line.

**Figure 5:**
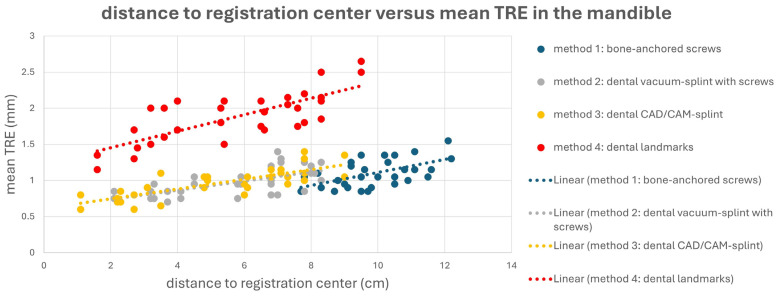
Point cloud plotting distance of the registration center in cm (*x* axis) versus mean target registration error (TRE) in mm (*y* axis) in the mandible for each registration method M1–M4. Regression line is displayed as dotted line.

This is also evident in Figure 4 and Figure 5. Here, a point cloud for each method is plotted separately for the midface and mandible, with the *x* axis showing the distance of the corresponding targets to the registration center of the respective method, and the *y* axis showing the value of the mean TRE. While the points and the regression line for M4 are significantly higher than those for the other methods, it is also noticeable that the slope of the regression line for M4 is steeper than that for the other methods, which again reflects the reduced reproducibility of finding the registration markers for M4. Particularly for methods M1 and M2, flat curve progressions are evident, which can partly be explained by the larger volume compared to the other methods, created by the four registration markers in space. However, the regression line for method M1 shows a greater slope in the mandible area, as all targets are relatively far from the registration center for M1.

**Figure 6:**
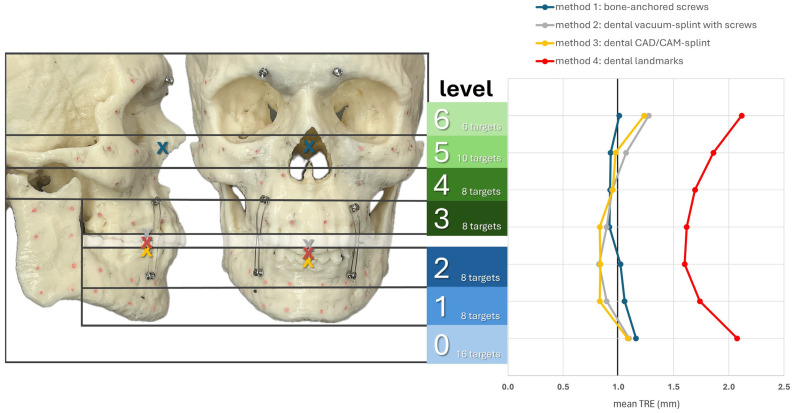
**Mean TRE in mm at each level of the facial skull for each registration method M1–M4.** Levels 0–2 group the targets across the mandible (0: ramus on both sides; 1: inferior mandibular border; 2: alveolar process of the mandible) while levels 3–6 group the targets across the midface (3: alveolar process of the maxilla; 4: maxillary sinus; 5: zygoma and infraorbital rim; 6: latero-orbital rim and posterior orbit). The registration center of each method is depicted as “X“ in the lateral and frontal view of the facial skull model.

Figure 6 shows the registration centers for the respective methods marked as an X on the skull model. The graph shows the respective TRE according to the level at which it was measured. The lowest TRE is shown at the height of the registration center for each method. Thus, methods M2–M4, which rely on the dental support of the registration markers, were most accurate at level 2, while M1, with a registration center at level 5, was most accurate at levels 5 and 4. For methods M2–M4, a significant increase in TRE was observed from level 6 onward, as the mean distance to the registration center was approximately 9 cm or more. In the area of the ramus (level 0), all methods resulted in a relatively high TRE. In particular, M1 showed its highest TRE value of 1.16 mm, as it also had the greatest distance to the registration center, with a value of 10.56 cm. Therefore, the two splint methods M2 and M3 showed relatively good values at level 0, with 1.10 mm for M2 and 1.08 mm for M3.

A multifactorial repeated measures analysis of variance (ANOVA) was performed (see Table 1) to determine whether the dependent variable TRE significantly differed across the groups defined by four factors: method, side, region, and level. The analysis also explored potential interactions between these factors. The ANOVA revealed significant differences in TRE for the factors ‘method’ (*p* < 0.001) and ‘level’ (*p* < 0.001), but no significant differences were observed for the other two factors (side: *p* = 0.759; region: *p* = 0.302). Significant interactions between the factors ‘method’ and ‘side’ (*p* < 0.001) and ‘method’ and ‘region’ (*p* < 0.027) were identified, while the interaction between ‘method’ and ‘level’ (*p* = 0.113) was not significant. Further analysis of the significant interactions (see Supplementary Tables S1 and S2) showed that mean TRE values ± standard deviation were lower on the right side compared to the left for methods M2 and M3, with statistical significance according to Student’s *t*-test (M2: *p* < 0.001, M3: *p* = 0.027). Moreover, mean TRE values ± standard deviation were lower in the midfacial region compared to the mandible for method M1 (*p* < 0.001).

Post hoc analysis using the Bonferroni correction (see Supplementary Table S3) revealed that method M4 had a significantly higher TRE compared to methods M1–M3 (*p* < 0.001), while no significant difference in TRE was observed between methods M1 and M3. For the factor ‘level’, levels 0 and 6 had significantly higher TREs than levels 1–4 (*p* < 0.001), but the difference was no longer significant when compared to level 5 (level 0 vs. 5: *p* = 0.04; level 6 vs. 5: *p* = 0.019).

Intra- and inter-rater reliability were calculated using intraclass correlation coefficients (ICCs) based on duplicate measurements across three run-throughs (twice by the first observer and once by the second observer) (see Table 2). The ICC values for intra-observer reliability were consistently close, ranging from 0.74 (moderate) for M1 to 0.80 (good) for M3. However, ICC values for interobserver reliability showed a wider distribution, with only M3 achieving a good result at 0.84, while the others were moderate, with M4 lagging significantly at 0.52. These results suggest that surgical navigation is subject to observer-dependent variability. Nonetheless, methods M1–M3 demonstrate acceptable reproducibility, with M3 achieving the highest reliability due to well-defined landmarks. In contrast, M4, which relies on dental landmarks, exhibits limitations in landmark definition, particularly in multi-observer scenarios, resulting in the lowest ICC of all four methods.

### 3.2. Surgical Cases

#### 3.2.1. Case 1: Secondary Zygoma and Orbital Floor Repair Following Trauma

**Figure 7:**
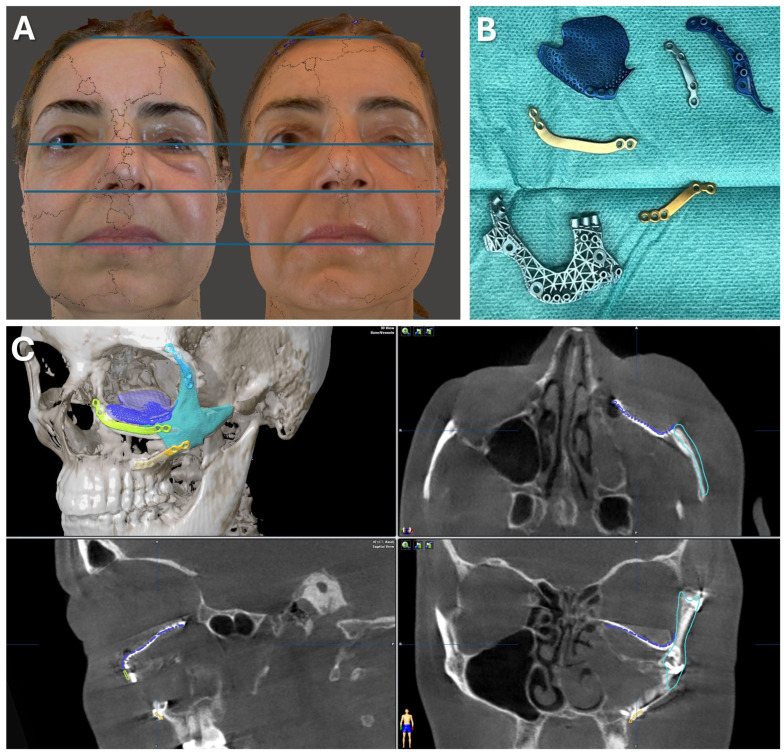
Case 1—Secondary repair of misplaced zygoma and orbital floor reconstruction. (**A**) Comparison of pre- and postoperative face scans shows that the asymmetry of the eyelids due to the hypoglobus and enophthalmos of the atrophic eyeball could be corrected. (**B**) Laser-sintered, color-coded patient-specific implants with corresponding drilling and cutting guides. (**C**) Although the guides could not be correctly positioned due to pseudarthrosis, the reconstruction results regarding the repositioned zygoma and the orbital implant placements were successful with accurate positioning due to intraoperative surgical navigation, which was proved by post-surgical analysis via the matching of the postoperative CBCT scan to the planning data shown in multi-planar view.

**Figure 8:**
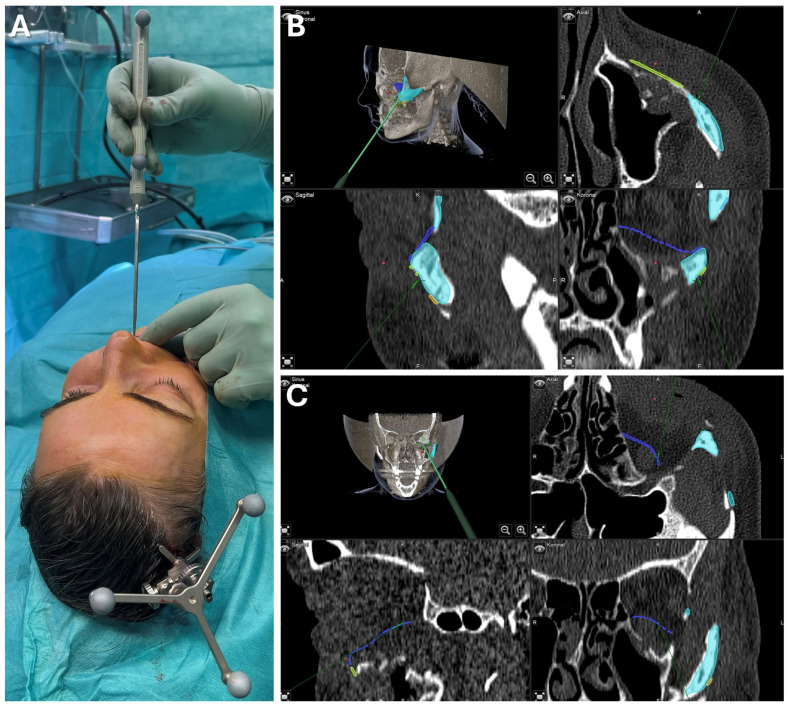
**Case 1—Secondary repair of misplaced zygoma and orbital floor reconstruction.** Facilitated by pointer-based surgical navigation utilizing the non-invasive registration protocol involving the CAD/CAM registration splint (**A**), an intraoperative assessment of zygoma repositioning was possible (**B**) before proceeding with the next step of orbital implant placement for orbital floor reconstruction, which could again be checked for accurate positioning via surgical navigation (**C**). Note the fixation of the skull reference array on the contralateral side of the affected zygoma visible in (**A**).

A 47-year-old patient presented with midfacial asymmetry due to the improper initial repositioning of an orbitozygomatic fracture following a bike accident 18 months prior (see Figure 7). Additionally, the patient exhibited phthisis bulbi as a result of a perforating globe injury. To restore facial symmetry, a one-stage surgical procedure was planned to reposition the zygomatic bone and reconstruct the orbital floor to match the contralateral side (see Figure 8). Subsequently, a scleral shell prosthesis was planned to camouflage the atrophic eye.

Patient-specific implants were used to restore the bony midface (Materialise NV, Leuven, Belgium). A mirrored DICOM dataset from a thin-slice CT scan was created using Mimics software (Materialise NV, Leuven, Belgium) to achieve symmetry. The zygomatic bone was virtually osteotomized, repositioned, and the orbital floor was re-contoured to match the healthy orbit. Polyamide cutting and drilling guides, along with titanium alloy implants, were designed and 3D-printed for use in surgery (see Figure 7). Due to the suspected pseudarthrosis in the zygoma area, surgical navigation was employed intraoperatively to verify the reconstruction outcome (see Figure 8). The DICOM data, STL files of the guides, implants, and repositioned zygoma were loaded into the navigation software (iPlan CMF 3.0.5, Brainlab AG, Feldkirchen, Germany). Registration was prepared using a dental CAD/CAM splint following the protocol presented herein, avoiding additional radiation exposure. Despite pseudarthrosis limiting the accurate placement of cutting guides, navigation confirmed the correct repositioning of the zygoma in sagittal and lateral dimensions (see Figure 8). This was critical for ensuring proper orbital rim reconstruction before proceeding with orbital floor repair.

Postoperative analysis confirmed the correct positioning of both the zygoma and orbital implant, even though the patient-specific plates did not perfectly align with the planned position (see Figure 7). Pre- and postoperative facial scans indicated that the reconstructed orbital floor adequately supported the orbital soft tissue, correcting hypoglobus, enophthalmos, and eyelid asymmetry (see Figure 7). This result provides an optimal foundation for the planned scleral shell prosthesis to restore overall facial symmetry in the next phase of treatment.

#### 3.2.2. Case 2: Mandibular Recontouring in a Case of Benign Central Osteoma

**Figure 9:**
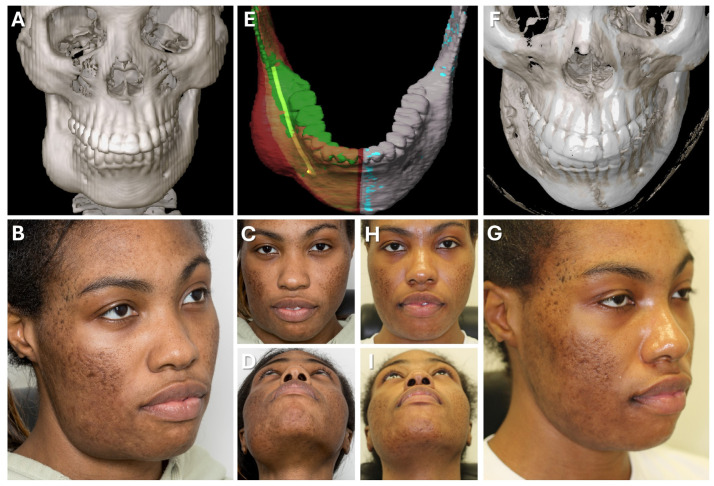
**Case 2—Mandibular Recontouring in a case of benign central osteoma.** Preoperative (**A**–**D**) and postoperative (**F**–**I**) radiological scans and clinical photographs are depicted. The virtual plan is shown in (**E**) with the segmented affected right side of the mandible (red) illustrating the bulge of the osteoma. The unaffected healthy left side (blue) was mirrored and aligned to the right side (green), while the mandibular canal was segmented in yellow to visualize the course of the inferior alveolar nerve during surgery.

A 26-year-old female patient presented with concerns about an unesthetic bulge on the right mandibular border, previously histologically confirmed as a benign central osteoma (see Figure 9). The condition was asymptomatic except for occasional mild to moderate pain, without sensory disturbance. Clinically, the patient was in good health, with no significant medical history aside from a third molar extraction on the right mandible three years earlier. Follow-up radiographs over two years showed a stable condition with no other osteomas present.

**Figure 10:**
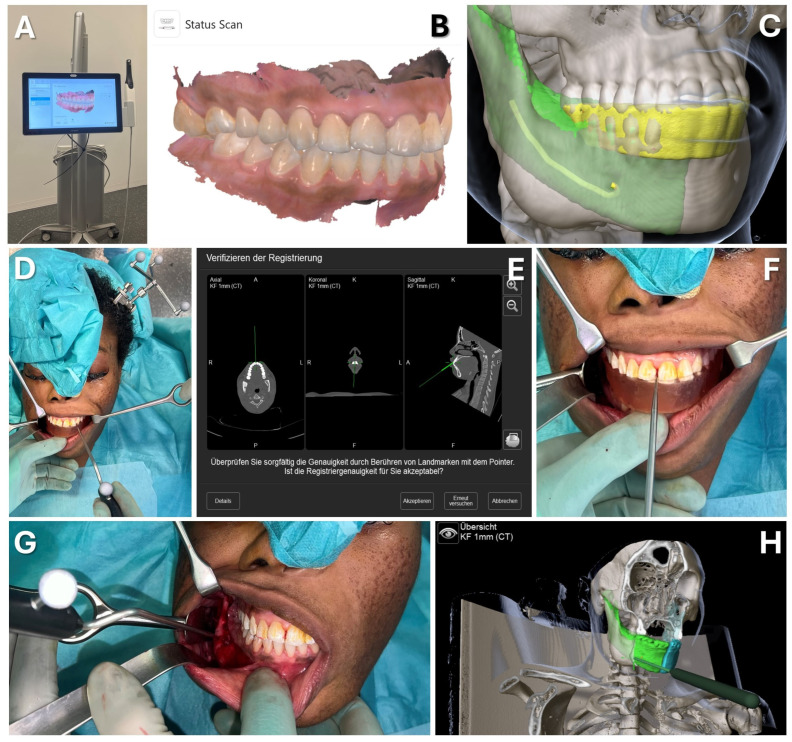
**Case 2—Mandibular Recontouring in a case of benign central osteoma.** Utilizing the TRIOS 3 from 3Shape (**A**) an intraoralscan was taken (**B**). This could be matched to the DICOM data along with virtual splint design, all made possible through the orthognathic planning software IPS Case designer from KLS Martin (not depicted, please refer to Figure 1). The virtual splint (yellow) can be loaded into the navigation software iPlan from Brainlab (**C**) to mark the registration landmarks in the splint‘s indentation (red). At the beginning of the surgery, the splint is put on to the upper dentition and registration landmarks are pointed at (**D**) until the system accepts the registration. After successful registration, results can be verified by checking the monitor (**E**) while pointing at anatomical landmarks (**F**). During the surgery, closing the mandible in habitual occlusion allowed for the use of navigation in the mandible (**G**), enabling the intraoperative verification of whether the virtually planned outcome had been achieved after recontouring (**H**).

Upon the patient’s request, osteoma reduction and mandibular recontouring were planned to enhance facial esthetics. The patient was informed about the risk of recurrence, and an intraoral approach was chosen to avoid external scarring due to her tendency for keloid formation. Due to the challenges of resecting in the submandibular and lingual areas through an intraoral route, surgical navigation was employed to overcome limited visibility. To avoid additional imaging, the newly developed registration protocol was used, incorporating the intraoral scans of both upper and lower dentitions in habitual occlusion, which was well defined and reproducible (see Figure 10).

For virtual surgical planning, the DICOM dataset from a thin-slice CT scan was imported and aligned to the Frankfurt horizontal and midsagittal plane, with the mandible in habitual occlusion. The healthy left mandibular side was mirrored and aligned with the right side, highlighting the excess bone from the osteoma. The mandibular canal was also segmented to assess the proximity to the inferior alveolar nerve during surgery. The CAD/CAM registration splint was designed for maxillary registration, enabling the navigation of the mandible once habitual occlusion was achieved intraoperatively.

Surgery was conducted using an intraoral approach, except for the skull reference array fixation on the contralateral side (see Figure 10). After registration, the bony reduction was guided by surgical navigation, ensuring the preservation of the inferior alveolar nerve and verifying the surgical outcome in real-time. Once a harmonious mandibular contour was achieved, navigation confirmed a sufficient resection according to the virtual plan, aiding in restoring facial symmetry (see Figure 10).

The clinical outcome (see Figure 9) was favorable, with the patient satisfied with the esthetic result. She retained full sensation after a brief period of hypoesthesia in the right inferior alveolar nerve and experienced pain relief.

## 4. Discussion

When it comes to surgical navigation, the registration process is crucial for ensuring accuracy in the operating room, which is key to the successful application of image-guided surgery. There are many different registration protocols, each with potential advantages and disadvantages in terms of performance, complexity, and invasiveness, among which the method based on bone-anchored fiducial markers is still considered as the gold standard with the highest accuracy for maxillofacial surgery [25]. However, usual protocols still rely on the invasive placement of these markers or the manufacturing of a dental splint, both requiring additional radiological imaging. This results in increased radiation exposure for the patient and organizational stress for the medical team due to the need for further scanning after the placement or integration of fiducial markers into the patient’s body.

A way of circumventing the invasive or non-invasive placement of actual fiducial markers and repeated imaging, while still enabling bony-based point-to-point registration, was first presented by Matsumoto et al. [32]. The authors described the virtual design of a surface-based template that fits the geometry of the mastoid. By placing holes into this template, the localization of virtually placed registration markers is guided, which allows the transfer onto the mastoid bone by drilling during the same surgery. These guided drill holes correspond to the virtual markers which therefore served as registration points during the setup of surgical navigation.

The same core principle applies to the protocol presented here. Although the dental splint does not directly guide the placement of bony registration markers, it incorporates these markers into its 3D-printed geometry as indentations. For this approach to work, the splint must fit precisely onto the teeth. This principle also governs the accuracy of splint-based jaw movements in virtual surgical planning for orthognathic surgery. Consequently, ensuring a precise fit of splints has been a focus of research in recent decades [33]. A critical factor is the detailed virtual patient model, which cannot be solely derived from CBCT or CT imaging due to the insufficient detail of the occlusal surface [34]. Therefore, composite models are used. Initially, conventional impressions and dental casts were scanned and overlaid, but advancements in the matching algorithm and intraoral scanning have enabled the more accurate integration of high-resolution dental geometry, simplifying the protocol and further improving accuracy [35]. With refinements in CAD/CAM procedures, such as offset calculations and advances in 3D printing, the precision of dental splints has been significantly enhanced [36,37]. Modern commercial orthognathic planning software now supports the entire workflow—from creating a composite virtual patient model to designing and fabricating a precisely aligned splint [38]. In our study, the correct orientation of the splint within the DICOM dataset is achieved through the matching algorithm of the IPS Case Designer. This rigid registration method is a combined surface-to-voxel approach which integrates the intraoral scan with the DICOM data with high precision, even in the presence of metal artifacts [39].

We evaluated the accuracy of the presented method in comparison with the standard methods of bone-anchored screws, dental vacuum-splint with screws, and dental landmarks in an ex vivo experimental setup involving a 3D-printed skull. We found that the presented method had a mean TRE of below 1 mm across the midface and mandible body. This new non-invasive CAD/CAM-splint-based method shows a comparable accuracy reflected in the observed low TRE to the bone-anchored screw method as well as the dental vacuum splint method, which represent the standard methods for surgical navigation in the maxillofacial region. Dental landmark registration was significantly less accurate in comparison.

Besides the evaluation of TRE, the fiducial localization error (FLE) is another established metric for measuring the accuracy of a registration method [22]. FLE is defined as the deviation between the position of a landmark (either anatomical or artificial fiducials) in the image data and its true physical location, and it can be a critical and limiting factor. The components contributing to this error can be subdivided into the following: (1) FLE_im_, the error in the identification or definition of landmarks in the image dataset; and (2) FLE_phy_, the error associated with pointing at the landmark with the navigation probe in the physical space during the intraoperative registration process. FLE_phy_ can be improved using landmarks with clearly defined geometries, such as holes, indentations, or screw heads, which facilitate correct probe positioning. This advantage applies to the new protocol as well as to screw methods (both bone-anchored and in the dental splint) due to the use of cross-slot or hexagon socket screw heads. In contrast, anatomical landmarks (whether bone or dental) typically only offer curved surfaces without distinct positioning features, which limits their effectiveness. The same issue affects FLE_im_, though metal artifacts also play a role here, as they can disrupt image data and significantly hinder the identification of even geometrically well-defined landmarks. Eckstein et al. interestingly found that FLE_im_ can be increased for both the bone-anchored screw method and the dental vacuum splint method, making an anatomical landmark approach even more accurate in some cases [30]. This increase in FLE_im_ is partly due to metal artifacts from dental restorations and the fiducial markers themselves, as well as the expertise of the planning surgeon [30]. Since the virtual splint is loaded as an STL file and thus unaffected by metallic artifacts, the precise definition of registration points at the indentations of the virtual splint should remain unhindered, even for inexperienced users.

The concept of the presented method was first described by Zeller et al. [40], emphasizing its application in maxillofacial traumatology. Schreurs et al. evaluated a similar method on cadavers and found comparably low TREs [41]. Instead of four indentations on the vestibular surface of the virtual splint, five extensions were applied, similarly to Zeller et al., who applied only four extensions. Theoretically, this would further increase the accuracy by increasing the number of registration markers and enlarging the distance between individual registration points. However, this results in a more complex virtual design, slightly extending the workflow, and the extensions could interfere with buccal soft tissues in certain patients during intraoperative integration, complicating the registration process. To achieve a vertical distribution of registration markers, the presented protocol follows an autorotation of the mandible during the CAD/CAM workflow. As the measured TREs of Schreurs et al. are comparable to the results reported here [42], it can be suggested that extensions may be omitted.

Besides navigation in the midfacial area, we evaluated the usage of the new method in the mandible and found it comparably accurate to the other bone-anchored methods as well. Limitations of this study include that the mandible in the initial DICOM dataset was already fixed, which would not be the case in a clinical scenario. However, in clinical application, it was shown that if the patient is in habitual occlusion during the initial CT scan, the method described here can still be applied. This is because the splint can be integrated into the virtual space despite the occlusion. This allows for registration to the maxilla with an open bite, and once the registration is completed, the bite can be closed to restore the corresponding situation from the initial CT scan. To ensure that the lower jaw is positioned according to the initial DICOM dataset during surgery, even if the patient does not offer a reproducible occlusion, an initial splint could also be generated by the IPS Case Designer software, which relates the mandible to the maxilla according to the DICOM dataset. Since no mandibular autorotation is applied in this scenario to achieve a greater vertical height of the splint, the application of vestibular extensions, as described by Zeller et al. [40] and Schreurs et al. [41], could enable the creation of a virtual splint with vertical registration marker distribution. This allows for accurate surgical navigation while maintaining the maxilla-to-mandible relationship as in the initial dataset. Thus, the virtual registration splint allows navigation not only in the midfacial region but also in the mandibular region. This was also proposed by Brouwer de Koning et al., although the virtual splint was not integrated via the surface matching of intraoral scans but rather by 3D-printing and radiological imaging with the printed splint in situ [43].

We believe that the new method, demonstrating low TRE values, is applicable to any area of maxillofacial surgery involving virtual planning, provided the patient has adequate dentition and intraoral scanning technology is accessible. While a learning curve is required, it should be manageable for those experienced in surgical navigation and CAD/CAM manufacturing. Table 3 outlines further limitations and advantages compared to standard methods. However, clinical studies are needed to assess whether TREs remain low during intraoperative use across various indications and whether enhancements in splint design could improve the surgical navigation accuracy. This study is the first to report the successful clinical application of this new registration protocol in the midface and mandibular regions, demonstrating high accuracy and clinical relevance for maxillofacial surgery.

## 5. Conclusions

The presented method of registration for surgical navigation offers a simple workflow that decreases patient radiation exposure and reduces organizational efforts for the surgical team, while still maintaining high accuracy with a TRE of ≤1 mm, which is comparable to the gold standard methods of point-based registration relying on bony or dental structures. The protocol can be applied not only to the midfacial region but also to the mandible, thereby making it applicable to a wide range of indications within the maxillofacial surgical spectrum. With new technologies like mixed-reality-driven navigation on the rise, which further expand the utilization of image-guided surgery, the need for a non-invasive yet accurate registration method is high. The presented and evaluated workflow can meet this need, making it valuable for future developments in computer-assisted surgery.

## Figures and Tables

**Table 1:** **Results of multifactorial ANOVA analysis.** *p*-values of *p* < 0.05 are considered statistically significant and are highlighted in bold text. df: degree of freedom; F: F-statistic; *p*: *p*-value; η^2^: eta-squared; η^2^_p_: partial eta-squared.

ANOVA		Sum of Squares	df	Mean of Squares	F	*p*	η^2^	η^2^_p_
A	Method (M1, M2, M3, M4)	68	3	22.67	241.71	**<0.001**	0.54	0.67
B	Side (right, left)	0.02	1	0.02	0.09	0.759	0	0
C	Region (midface, mandible)	0.17	1	0.17	1.07	0.302	0	0.01
D	Level (0, 1, 2, 3, 4, 5, 6)	7.96	6	1.33	12.87	**<0.001**	0.06	0.39
A × B	Method × side	2.28	3	0.76	8.41	**<0.001**	0.02	0.06
A × C	Method × region	0.87	3	0.29	3.09	**0.027**	0.01	0.02
A × D	Method × level	2.42	18	0.13	1.43	0.113	0.02	0.07

**Table 2:** **Intraclass correlation coefficient.** ICCs ranging from 0 to 1, with values <0.5 considered as poor, 0.5–0.75 as moderate, 0.75–0.90 as good, and >0.90 as excellent in terms of agreement/reliability. CI: confidence interval; F: F-statistic; df1: degree of freedom 1; df2: degree of freedom 2; *p*: *p*-value.

Intraclass Correlation Coefficient		ICC	95% CI Lower Limit	95% CI Upper Limit	F	df1	df2	*p*
**Intraobserver**	Method 1: bone-anchored screws	0.74	0.66	0.81	6.78	127	127	<0.001
	Method 2: dental vacuum-splint with screws	0.79	0.72	0.85	8.69	127	127	<0.001
	Method 3: dental CAD/CAM-splint	0.80	0.73	0.86	8.99	127	127	<0.001
	Method 4: dental landmarks	0.78	0.70	0.84	8.10	127	127	<0.001
**Interobserver**	Method 1: bone-anchored screws	0.71	0.61	0.79	5.85	127	127	<0.001
	Method 2: dental vacuum-splint with screws	0.69	0.59	0.77	5.50	127	127	<0.001
	Method 3: dental CAD/CAM-splint	0.84	0.79	0.89	11.89	127	127	<0.001
	Method 4: dental landmarks	0.52	0.38	0.64	3.17	127	127	<0.001

**Table 3:** Advantages and limitations of the presented registration protocol.

Advantages	Limitations
High accuracy in maxillofacial region	Not applicable in cases with significant injury to or defect of the dentition/maxilla or in cases with severe jaw clamp
High patient comfort	Adequate dentition needed
No additional radiation exposure	Dentition must be fully represented in the DICOM dataset
Reduced preparation time and organization effort	Needs 3D printing and intraoral scanning devices which may pose further costs, if not readily available at clinical site
Reproducible and applicable splint fabrication in case of loss or damage	Learning curve may be needed for implementation in clinical practice

## Data Availability

Dataset available on request from the authors.

## Supplementary Materials

The following supporting information can be downloaded at: https://www.mdpi.com/article/10.3390/jcm13175196/s1, Table S1: Results of Student’s *t*-test for the analysis of interaction between method and side, Table S2: Results of Student’s *t*-test for the analysis of interaction between method and region, Table S3: Results of Bonferroni post hoc tests.
