# Supplementary material for: Intraoral Scanning Enables Virtual-Splint-Based Non-Invasive Registration Protocol for Maxillofacial Surgical Navigation
Source: J Clin Med. 2024 Sep 2;13(17):5196. doi: 10.3390/jcm13175196 (PMC11396243; doi:10.3390/jcm13175196)
Supplement: Supplementary file 1 [file jcm-13-05196-s001.zip › jcm-3181616-supplementary.pdf]

# Supplementary material

**Supplementary Table S1: Results of Student's t-test for analysis of interaction between method and side.** P-values of $p < 0.05$ are considered statistically significant and are highlighted in bold text. SD: standard deviation; CI: confidence interval; t: t-statistic; df: degree of freedom; p: p-value; Cohens d: measure of effect size

| Student's t-test | side | mean | SD | mean differ-ence | 95% CI lower limit | 95% CI upper limit | t | df | p | Cohens d |
|---|---|---|---|---|---|---|---|---|---|---|
| Method 1: bone-anchored screws | r | 1.02 | 0.21 | 0 | -0.09 | 0.08 | -0.11 | 126 | 0.909 | 0.02 |
| | l | 1.02 | 0.25 | | | | | | | |
| Method 2: dental vacuum-splint with screws | r | 0.89 | 0.3 | -0.23 | -0.34 | -0.12 | -4.2 | 126 | **<0.001** | 0.74 |
| | l | 1.12 | 0.32 | | | | | | | |
| Method 3: dental CAD/CAM-splint | r | 0.91 | 0.31 | -0.12 | 0.01 | 0.22 | 2.24 | 126 | **0.027** | 0.4 |
| | l | 1.03 | 0.27 | | | | | | | |
| Method 4: dental landmarks | r | 1.88 | 0.46 | 0.07 | -0.08 | 0.23 | 0.95 | 126 | 0.344 | 0.17 |
| | l | 1.8 | 0.44 | | | | | | | |

**Supplementary Table S2: Results of Student's t-test for analysis of interaction between method and region.** P-values of $p < 0.05$ are considered statistically significant and are highlighted in bold text. SD: standard deviation; CI: confidence interval; t: t-statistic; df: degree of freedom; p: p-value; Cohens d: measure of effect size

| Student's t-test | region | mean | SD | mean differ-ence | 95% CI lower limit | 95% CI upper limit | t | df | p | Cohens d |
|---|---|---|---|---|---|---|---|---|---|---|
| Method 1: bone-anchored screws | midface | 0.94 | 0.19 | -0.16 | -0.23 | -0.08 | -4.11 | 126 | **<0.001** | 0.73 |
| | mandible | 1.10 | 0.24 | | | | | | | |
| Method 2: dental vacuum-splint with screws | midface | 1.03 | 0.41 | 0.05 | -0.06 | 0.17 | 0.94 | 97.43 | 0.35 | 0.17 |
| | mandible | 0.98 | 0.22 | | | | | | | |
| Method 3: dental CAD/CAM-splint | midface | 0.98 | 0.35 | 0.02 | -0.08 | 0.13 | 0.42 | 110.49 | 0.678 | 0.07 |
| | mandible | 0.96 | 0.23 | | | | | | | |
| Method 4: dental landmarks | midface | 1.81 | 0.5 | 0.07 | -0.22 | 0.09 | -0.83 | 126 | 0.408 | 0.15 |
| | mandible | 1.87 | 0.39 | | | | | | | |

**Supplementary Table S3: Results of Bonferroni post hoc tests.** Corrected p-values of p < 0.008 for methods, p < 0.05 for side and region and p < 0.002 for level are considered statistically significant and are highlighted in bold text. t: t-statistic; p: p-value.

| Bonferroni post hoc tests | | mean difference | standard error | t | p |
|---|---|---|---|---|---|
| *Methods* | | | | | |
| M1 | M2 | 0.01 | 0.034 | 0.412 | 1 |
| M1 | M3 | 0.05 | 0.032 | 1.521 | 0.784 |
| M1 | M4 | -0.82 | 0.04 | -20.476 | **<0.001** |
| M2 | M3 | 0.04 | 0.036 | 0.968 | 1 |
| M2 | M4 | -0.83 | 0.046 | -18.051 | **<0.001** |
| M3 | M4 | -0.87 | 0.041 | -21.04 | **<0.001** |
| *Side* | | | | | |
| right | left | -0.01 | 0.04 | -0.31 | 0.759 |
| *Region* | | | | | |
| midface | mandible | -0.04 | 0.04 | -1.04 | 0.302 |
| *Level* | | | | | |
| 0 | 1 | 0.22 | 0.05 | 4.56 | **<0.001** |
| 0 | 2 | 0.28 | 0.05 | -5.77 | **<0.001** |
| 0 | 3 | 0.29 | 0.05 | -5.86 | **<0.001** |
| 0 | 4 | 0.23 | 0.05 | -4.59 | **<0.001** |
| 0 | 5 | 0.15 | 0.05 | -3.17 | 0.04 |
| 0 | 6 | -0.05 | 0.05 | 1 | 1 |
| 1 | 2 | 0.06 | 0.06 | -1.05 | 1 |
| 1 | 3 | 0.06 | 0.06 | -1.13 | 1 |
| 1 | 4 | 0 | 0.06 | -0.03 | 1 |
| 1 | 5 | -0.08 | 0.05 | 1.47 | 1 |
| 1 | 6 | -0.28 | 0.06 | 4.55 | **<0.001** |
| 2 | 3 | 0 | 0.06 | -0.08 | 1 |
| 2 | 4 | -0.06 | 0.06 | 1.02 | 1 |
| 2 | 5 | -0.14 | 0.05 | 2.57 | 0.239 |
| 2 | 6 | -0.34 | 0.06 | 5.51 | **<0.001** |
| 3 | 4 | -0.06 | 0.06 | -1.1 | 1 |
| 3 | 5 | -0.14 | 0.05 | -2.66 | 0.187 |
| 3 | 6 | -0.34 | 0.06 | -5.59 | **<0.001** |
| 4 | 5 | -0.08 | 0.05 | -1.5 | 1 |
| 4 | 6 | -0.28 | 0.06 | -4.57 | **<0.001** |
| 5 | 6 | -0.2 | 0.06 | -3.4 | 0.019 |
